# ENDOSCOPIC CHANGES RELATED TO GASTROESOPHAGEAL REFLUX DISEASE: COMPARATIVE STUDY AMONG BARIATRIC SURGERY PATIENTS

**DOI:** 10.1590/S0102-6720201500S100011

**Published:** 2015-12

**Authors:** Marco Aurelio SANTO, Sylvia Regina QUINTANILHA, Cesar Augusto MIETTI, Flavio Masato KAWAMOTO, Allan Garms MARSON, Roberto de CLEVA

**Affiliations:** Bariatric and Metabolic Surgery Unit, Digestive Surgery Discipline, Department of Gastroenterology, Hospital das Clinicas, Medical School, University of São Paulo, São Paulo, SP, Brazil

**Keywords:** Morbid obesity, Gastroesophageal reflux, Esophagitis, Hiatal hernia, Bariatric surgery

## Abstract

***Background*:**

Obesity is correlated with several comorbidities, including gastroesophageal reflux disease. Its main complications are detectable by endoscopy: erosive esophagitis and Barrett's esophagus.

**Aim:**

: To correlate erosive esophagitis and hiatal hernia with the degree of body mass index (BMI).

**Method:**

: Was performed a retrospective analysis of 717 preoperative endoscopic reports of bariatric patients. Fifty-six (8%) presented hiatal hernia, being 44 small, nine medium and five large. Esophagitis was classified by Los Angeles classification.

**Results:**

: There was no correlation between the presence and dimension of hiatal hernia with BMI. One hundred thirty-four (18.7%) patients presented erosive esophagitis. Among them, 104 (14.5%) had esophagitis grade A; 25 (3.5%) grade B; and five (0.7%) grade C. When considering only the patients with erosive esophagitis, 77.6% had esophagitis grade A, 18.7% grade B and 3.7% grade C. Were identified only two patients with Barrett's esophagus (0,28%).

**Conclusion:**

: There was a positive correlation between the degree of esophagitis with increasing BMI.

## Abstract (pt)

***Racional*:**

A obesidade está correlacionada com diversas comorbidades, dentre elas a doença do refluxo gastroesofágico. Ela tem como um de seus principais desencadeantes a hérnia do hiato, e como suas principais complicações a esofagite erosiva e o esôfago de Barrett.

**Objetivo:**

: Correlacionar o grau do índice de massa corporal (IMC) com a presença e tamanho da hérnia hiatal, e com a presença e gravidade da esofagite erosiva e esôfago de Barrett.

**Método:**

: Foi realizada análise retrospectiva de laudos endoscópicos pré-operatórios de 717 pacientes submetidos à cirurgia bariátrica. A hérnia de hiato esteve presente em 56 pacientes (8%), sendo que delas 44 eram pequenas, nove médias e cinco grandes. O grau da esofagite obedeceu o preconizado pela Classificação de Los Angeles.

**Resultados:**

: Não houve correlação entre a presença ou tamanho da herniação hiatal com o IMC. Dos pacientes avaliados, 134 (18,7%) apresentavam esofagite erosiva. Dentre elas 104 (14,5%) eram grau A; 25 (3,5%) grau B e cinco (0,7%) grau C. Considerando-se apenas os portadores de esofagite erosiva, 77,6% eram grau A; 18,7% grau B; e 3,7% grau C. Foram identificados apenas dois casos de esôfago de Barrett (0,28% da amostra total).

**Conclusão:**

: Observou-se correlação positiva entre o grau de esofagite com o aumento do IMC.

## INTRODUCTION

The World Health Organization defines obesity as fat accumulation that determines health risk. Body mass index (BMI) was established as a worldwide standard for assessing the severity of obesity, which is calculated by dividing the patient's weight in kilograms by the square of their height in meters. Presence of obesity is considered when BMI>30 kg/m². Patients with IMC≥35 and <40 kg/m^2^are classified with class II obesity; with BMI>40 kg/m² grade III or serious; and BMI>50 kg/m² super-obese.

In the United States it is estimated that one third of the adult population is in the obesity range and 4.8% of the population over 20 years present morbid obesity^1^. In São Paulo the percentage of obese adults is approximately 12%, with higher prevalence in young adult population. Obesity is accompanied by associated systemic diseases such as hypertension, diabetes mellitus, insulin resistance, dyslipidemia and diseases of the digestive tract such as gastroesophageal reflux disease (GERD), cholelithiasis and non-alcoholic fatty liver disease.

Symptomatic GERD is frequent in the population of obese patients, with prevalence ranging from 30-60%^5^. Patients with obesity have a high intra-abdominal pressure and consequent increase in gastroesophageal pressure gradient, increasing both the esophageal gastric juice exposure^6^ juice, and the risk of hiatal hernia^8^. Apart from GERD and erosive esophagitis, recent studies show increased incidence of esophageal adenocarcinoma in morbid obese^11^^,^ 12. However, there is no description in the literature about the relationship of the occurrence of these changes with the strongest degrees of severe obesity.

The intense and prolonged exposure of the esophageal epithelium to gastric juice causes chronic esophagitis and in the damaged can be observed replacing of the squamous epithelium columnar cells by intestinal metaplasia, condition called Barrett's esophagus. This metaplastic process can progress to dysplastic process and subsequent formation of adenocarcinoma^25^. The traditional definition of Barrett's esophagus required the metaplastic epithelium be over the extent to 3 cm from the esophagogastric junction. However, more recently it was observed that lesions with involvement of lower extension of the distal esophagus mucosa, and even restricted to the esophagogastric junction, are related to gastroesophageal reflux and have malignant potential, and therefore are also classified as Barrett's esophagus^3^.

The aim of this study was to evaluate the endoscopic GERD-related changes in the preoperative of bariatric surgery, comparing the degree of BMI and the prevalence of hiatal hernia, erosive esophagitis and Barrett's esophagus.

## METHODS

Were studied 717 patients undergoing bariatric surgery at the Bariatric and Metabolic Surgery Unit of the Hospital das Clinicas, Faculty of Medicine, University of São Paulo, São Paulo, SP, Brazil, from 2007 to 2012.

It was a retrospective study with analysis of preoperative endoscopic reports and evaluated the changes related to GERD (hiatal hernia, reflux esophagitis and Barrett's esophagus) as classified below.

Hiatus hernia was classified according to the size of the herniated gastric chamber: 1) small, 1 to 3 cm; 2) medium, between 3 cm and 5 cm; and 3) large, more than 5 cm.

**FIGURE 1:**
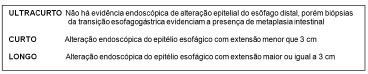
- Barrett's esophagus classified in accordance to its extension

Reflux esophagitis was classified according to the Los Angeles classification. Barrett's esophagus was classified according to their length (Figure 1).

Patients were divided into three groups according to BMI: group I, BMI ≥35 and <40 kg/m^2^; group II, IMC≥40 and <50 kg/m^2^; and group III, IMC≥50.

### Statistical analysis

It was performed using the SPSS 12 (SPSS, Chicago, Illinois). The data of continuous variables were expressed as mean±standard deviation and categorical variables as percentages. The differences between the groups in continuous variables were determined using the Student t test and the categorical variables using Chi-square, and the relationship between severity of esophagitis and BMI was given by Gamma test, with the level of defined statistical significance at p<0.05.

## RESULTS

### Hiatal hernia

The analysis showed the presence of endoscopic hiatal hernia in 8% of patients (n=58) whereas in 44 it was small, nine medium and five large (Table 1).

No correlation was observed between the presence of hernia and BMI (p=0.612). There was no positive correlation between the presence or size of hiatal hernia between super-obese (BMI>50), patients with a BMI between 40 and 50 and patients with BMI between 35 and 40 (obesity GII).

**TABLE 1:** - Distribution in number and percentage of patients with hiatal hernia by the size

**Herniation**	**n**	**% sample**	**Hernia**
Small	44	6%	76%
Medium	9	1.3%	15%
Large	5	0.7%	9%
TOTAL	58	8%	100%

### Reflux esophagitis

It was observed the presence of reflux esophagitis in 134 patients, corresponding to 18.7% of the sample (n=717). Compared to the total sample 14.5% (n=104) had erosive esophagitis grade A; 3.5% (n=25) grade B; and 0.7% (n=5) grade C. No patient had esophagitis grade D. Considering only patients with esophagitis (n=134), 77.6% had esophagitis grade A, 18.7% grade B and 3 7% grade C (Table 2).

**TABLE 2:** - Distribution in number and percentage of patients with reflux esophagitis by Los Angeles classification

**Classification**	**n**	**% sample**	**DRGE**
Grade A	104	14.5%	77.6%
Grade B	25	3.5%	18.7%
Grade C	5	0.7%	3.7%
Grade D	0	0%	0%
TOTAL	134	18,7%	100%

Classifying patients with esophagitis according to BMI, it was observed that nine had BMI between 35 and 40, 79 between 40 and 50, and 46 were superobese. (Table 3)

**TABLE 3:** - Distribution of patients by BMI and the presence/severity of reflux esophagitis

**BMI**	**n**	**Esophagitis**	**Grade A**	**Grade B**	**Grade C**
≥35 e <40	81	9 (11.1%)	6 (7.4%)	3 (3.7%)	0
≥40 and <50	435	79 (18.1%)	61 (14.0%)	14 (3.2%)	4 (1%)
≥50	201	46 (22.8%)	37 (18.4%)	8 (3.9%)	1 (0.5%)
TOTAL	717	134	104	25	5

There was a positive correlation between the presence of erosive esophagitis and BMI. Superobese patients had a higher prevalence of esophagitis than obese with BMI between 35 and 40 (obesity GII) (p=0.03). Comparing superobese and patients with BMI between 40 and 50 was not identified any relationship (p=0.165), also in patients with BMI between 35 and 40 (obesity GII) and BMI between 40 and 50 (p = 0.148). 

### Barrett's esophagus

It was observed only two cases of Barrett's esophagus in 717 endoscopies, showing prevalence of 0.28% in the sample.

## DISCUSSION

Currently, the prevalence of GERD has increased, affecting between 8-26% of the occidental population^26^. Associated with the increase, was observed increase in related complications, including Barrett's and adenocarcinoma of the esophagus^4^. Reasons for both increase in GERD, as its complications, are not yet completely understood.

It should be noted that the increased prevalence of GERD accompanies the worldwide epidemic obesity. The effect of weight gain on the GERD is important, it is estimated that the increase of 3.5 points in BMI increases by approximately three times the risk of developing symptoms of reflux^21^.

Obesity has also been associated with increased intra-abdominal pressure^2^, decrease in gastric emptying^18^ and in lower esophageal sphincter pressure, and increase of transient sphincter relaxation^23^, changes that together determine increased esophageal exposure to hydrochloric acid.

Some papers demonstrated the association of GERD with the prevalence of obesity, especially the ones of Nilsson^22^, Murray^20^ and Lagergren^17^. It should be noted that no studies have observed negative association between GERD and obesity.

In this paper, 18.7% of obese patients had esophagitis, prevalence greater than that observed in general population^14^, suggesting a positive association between them, although the prevalence is similar to that observed in occidentals^26^. There was a positive correlation between the presence of erosive esophagitis and BMI, particularly when compared superobese patients and patients with BMI between 35 and 40 (obesity GII) (p=0.03). Comparing superobese and patients with BMI between 40 and 50 was not identified this relationship (p=0.165), as far as between patients with BMI between 35 and 40 (obesity GII) and BMI between 40 and 50 (p=0.148). Thus, the presence of more severe obesity is considered as a risk factor for esofagitis^27^.

Both overweight and obesity meet numerous criteria for association with GERD, including hiatal hernia. Obese patients are at increased risk for hiatal hernia, which is one of the factors associated with DRGE^7^^,^ 28.

In this study, although there were hiatal hernia in all obese groups, it was not possible to correlate its presence with the progressive increase in BMI.

Moreover, while weight loss is often recommended as a therapy for improving reflux disease^15^, many studies are contradictory regarding its efficacy^9^^,^ 16. Small non-randomized studies suggest that weight loss after bariatric surgery may be associated with improvement of symptoms of reflux^10^^,^ 13, although none suggests that weight loss reduces the risk of esophageal adenocarcinoma. In this series, only were identified two patients with Barrett's esophagus, and no cases of esophageal adenocarcinoma.

Although positive association was identified between the presence of obesity and GERD, it would be expect that such association should be more significant, which may suggest another paradox of obesity^24^, mainly because GERD itself affects 6-22% of the occidental population^19^. However, highlights there are endoscopically diagnosed change, which features GERD as a significant comorbidity in the context of diseases associated with obesity. 

## CONCLUSION

There was a positive correlation between the degree of esophagitis with the BMI increase. 
